# Clinical yarning with Aboriginal and/or Torres Strait Islander peoples—a systematic scoping review of its use and impacts

**DOI:** 10.1186/s13643-022-02008-0

**Published:** 2022-06-23

**Authors:** Alexander W. Burke, Susan Welch, Tamara Power, Cherie Lucas, Rebekah J. Moles

**Affiliations:** 1 Sydney Pharmacy School, Faculty of Medicine and Health, The University of Sydney, Pharmacy Building A15, Science Road, Camperdown, NSW 2006 Australia; 2 Pharmacy Department, St Vincent’s Hospital, Darlinghurst, NSW 2010 Australia; 3 Susan Wakil School of Nursing, Faculty of Medicine and Health, The University of Sydney, Camperdown, NSW 2006 Australia; 4 Graduate School of Health, University of Technology Sydney, Ultimo, NSW, 2007 Australia

**Keywords:** Aboriginal and/or Torres Strait Islander, Yarning, Clinical yarning, Health outcomes

## Abstract

**Objectives:**

To explore how clinical yarning has been utilised as a health intervention for Aboriginal and/or Torres Strait Islander peoples and if there are any reported impacts yarning might have on health outcomes.

**Study design:**

Systematic scoping review of published literature.

**Data sources:**

A one-word search term “yarning” was applied in Scopus, EMBASE, CINAHL, MEDLINE, International Pharmaceutical Abstracts, Australian Public Affairs Information Service-Health, and the Aboriginal and/or Torres Strait Islander Health Bibliography databases. Databases were searched from inception to May 20, 2020.

**Study selection:**

Studies were included where clinical yarning had been used as a health intervention. Inclusion and exclusion criteria were developed and applied according to PRISMA systematic and scoping review reporting methods.

**Data synthesis:**

A total of 375 manuscripts were found from the initial data search. After removal of duplicates and removal of manuscripts based on abstract review, a total of 61 studies underwent full-text review. Of these, only five met the inclusion criteria of utilising yarning as a clinical intervention. Four of these studies described consumer self-reported health outcomes, with only one study looking at improvements in objective physiological health outcomes.

**Conclusions:**

Whilst clinical yarning may be a culturally appropriate intervention in healthcare, there are limited studies that have measured the impact of this intervention. Further research may be needed to ascertain the true benefits of this intervention.

## Background

Aboriginal and/or Torres Strait Islander peoples in Australia belong to the world’s oldest continuing cultures. As a direct consequence of colonisation, Aboriginal and Torres Strait Islander peoples face far worse health outcomes than the broader population of the nation [1]. The gap in life expectancy is 8 years less than the national average with two thirds of Aboriginal and/or Torres Strait Islander peoples dying before the age of 65 years [2]. This has been an ongoing problem, with various strategies put in place to try to improve the gap that exists between Aboriginal and Torres Strait Islander peoples and the rest of Australia. The Closing the Gap (CTG) strategy has been in place since 2007, where Australian governments have worked together to deliver better health, education and employment outcomes [1]. Despite these efforts, recent reports still estimate that the targets for reducing the gap in mortality will not be met by 2031 [1].

Issues impacting the health of Aboriginal and/or Torres Strait Islander peoples include having higher rates of non-communicable diseases, as well as increased disadvantage and lower levels of education [1]. With respect to health, they are 2 times more likely to have a myocardial infarction compared to the standard population, 1.2 times more likely to have hypertension and 4 times more likely to have type 2 diabetes [1, 3]. These statistics are important, because any change that can potentially improve health outcomes for Aboriginal and/or Torres Strait Islander peoples should be considered as a serious alternative to the systems currently in place. Providing healthcare to Aboriginal and/or Torres Strait Islander peoples should ensure a holistic approach that is provided in a culturally safe and appropriate manner [4]. One of the techniques that is considered culturally appropriate is clinical yarning [4].

Before a more in-depth look at what clinical yarning is, a general look at what the term yarning means to Aboriginal and/or Torres Strait Islander Australians is important. Yarning is a conversation that involves the sharing of one’s own stories and the creation of new knowledge [4]. It prioritises Aboriginal and/or Torres Strait Islander ways of communicating, in that it is culturally appropriate and respectful [5]. Yarning has a special place in Aboriginal culture, and the practice has been around for millennia. Yarning involves a 2-way dialogue of sharing and receiving information between people that is built on the relationship that the parties involved have with each other, certain cultural protocols that should be followed and respects what each person wishes to get out of the dialogue [4]. It should be noted that this is however a general definition, and it is hard to make an accurate description of exactly what yarning is, as it can be applied differently from person to person and even have different application across Aboriginal nations in Australia [4].

Due to the long history of the use of yarning as a culturally safe form of communication between Aboriginal and/or Torres Strait Islander Australians, there recently has been a switch to yarning-based communication for research and therapies for Aboriginal and/or Torres Strait Islander populations [5]. The idea behind this move to approach research and health in the framework of the yarn is to hopefully result in more accurate portrayal of Aboriginal and/or Torres Strait Islander perspectives compared to standard closed-style questioning [5]. Closed-style questioning could also be confrontational to an Aboriginal and/or Torres Strait Islander person and trying to further develop a relationship when this barrier has been formed can be difficult [5]. In this way, it is apparent that clinical yarning may be aligned with patient-centred care and shared decision making principles, allowing opportunities for a less paternalistic approach to healthcare [5].

Yarning in a clinical setting has three interrelated areas that are recommended in order to engage a patient in their healthcare journey. The first involves the “social yarn” where one tries to find common ground with the patient. This first part of the yarn is the steppingstone for applying the other two areas of the clinical yarn [5]. The second dimension of the clinical yarn is known as the “diagnostic yarn” where the diagnostician encourages the patient to tell their health story which means the patient might describe in detail the events about their life that may relate to the patient’s present medical conditions. This is best performed as an open-ended dialogue where the practitioner will unpack the relevant pieces of information and apply it to their own knowledge which will inform their decision about how to best manage the condition the patient has presented with [5]. Finally, the “management yarn” is implemented. In this stage, the practitioner will provide straight forward information to the patient but may use metaphors and stories connected to the patient’s life to make it easier for the patient to understand the condition they have. Also, by involving the patient in the decision-making process they may become more motivated in their own health and regain their autonomy. Regaining autonomy has been cited as being a key part of the clinical yarn, as many Aboriginal and/or Torres Strait Islander peoples believe their autonomy has been stripped from them since the time of colonisation [5].

Other countries with Indigenous populations have similar interventions that Australia could integrate into its healthcare system. The Native American people have a similar concept to yarning groups called “Talking circles” [6]. Talking circles are a traditional way that Native Americans come together to communicate and solve problem. In the circle, people are given a voice to express themselves freely and are empowered to have a voice and feel heard and supported [6]. A study by Nadeau et al. looked at the implementation of monthly 2-h talking circles with other interventions to get Native elders to talk about tobacco use and their beliefs and perceptions with it [7]. They found that from these interventions, elder knowledge about commercial tobacco products was increased and the elders who took part believed that the implementation of the talking circles was effective [7]. Another study conducted by Wilken and Nunn, looked at the effect talking circles may have on medication adherence [8]. They found that although more studies are needed in the area, talking circles may have an impact in improving medication adherence in Native Americans with uncontrolled type 2 diabetes [8].

The studies mentioned did show that Indigenous focused communication methods and using it as a clinical tool may lead to improvements in Indigenous patient outcomes. By implementing a clinical yarning approach to Aboriginal and/or Torres Strait Islander health care, there could be benefits associated with it that are not seen within the conventional healthcare system. It has been stated that the conventional system is often seen as a barrier to improving health outcomes for Aboriginal and/or Torres Strait Islander peoples [5]. Issues include lack of the use of Indigenous languages, the use of medical jargon and the clinical approach to providing “Western” healthcare [5]. These issues can cause Aboriginal and/or Torres Strait Islander peoples to feel alienated from their healthcare and make them disengage from the healthcare system [5]. Generally, Aboriginal and/or Torres Strait Islander peoples do want to be involved with their healthcare [5, 9]; however, the information is often presented in a way that is incongruent with Aboriginal and/or Torres Strait Islander peoples’ beliefs about health, making it harder for them to connect [5, 9].

Using clinical yarning as a framework may make conversing with Aboriginal and/or Torres Strait Islander peoples more accessible and meaningful and potentially may have positive impacts on health outcomes contributing to lessening the health disparity gap. This review therefore questioned “how has clinical yarning has been utilised as a health intervention for Aboriginal and/or Torres Strait Islander Peoples?” and “what are the impacts of yarning on health outcomes?” By answering these questions, we may be able to make inference as to whether health outcomes may be better achieved using traditional communication techniques than through western styles of health communication.

## Method

### Search strategy

A single-word search strategy was used—“yarning”. This single term was chosen because yarning is a unique word to explain conversation within the Aboriginal and/or Torres Strait Islander context. “Clinical yarning” due to being a new and developing concept was not used as the search term as it was perceived that this may be too narrow to pick up the relevant studies. The term “yarning” was therefore entered as a keyword search term into seven databases. Databases included Scopus, EMBASE, CINAHL, MEDLINE, International Pharmaceutical Abstracts (IPA), Australian Public Affairs Information Service (APAIS)-Health and the Aboriginal and/or Torres Strait Islander (ATSI)-Health Bibliography. The database searches were conducted between March 2020 and May 2020 and identified published publications from database inception up until May 20, 2020. The PRISMA systematic review reporting method was used to collate the data obtained [10]. Details of the search strategy and yields are tabulated in Appendix 1.

### Eligibility criteria

Only manuscripts written in English, reporting on primary research outcomes, were included for review. Hence, conference abstracts, editorials, commentaries, opinion articles and other literature reviews were excluded. Studies were excluded if the manuscript did not pertain to yarning for health and if yarning was used only as a data collection tool rather than a health intervention. If yarning was use as both a data collection tool AND a health intervention, the manuscript was included. Studies were also excluded if yarning was reported only as an important outcome of how healthcare should be delivered. For example, if yarning was considered a useful method to convey health information however it was not actually used as the health intervention itself, the manuscript was excluded. Table 1 shows all inclusion and exclusion criteria applied.

**Table 1:** Inclusion/exclusion criteria

Inclusion	Exclusion
Written in English	Not written in English
Publication reporting on primary research outcomes	Conference abstract, editorials, commentaries, opinion articles and other literature reviews
Yarning was used as a health intervention	Yarning not in the context of health
Australian	Not Australian
	Yarning only used for data collection
	Yarning mentioned as a way healthcare should be delivered but not used as an intervention

### Study selection and data extraction

The searches were undertaken by one author (AB) by using the agreed upon inclusion/exclusion criteria. Validation of search results was conducted by another author (RM), who undertook independent searches in three of the seven databases with the same yields identified. Search results from all databases were exported into EndNote [11] where duplicates were removed. Titles and article types were then screened by the lead author (AB), followed by an abstract review. Full-text review followed and was conducted by two authors, and where there was any ambiguity over a publication’s inclusion or exclusion, a discussion by two authors (RM and AB) occurred to reach consensus. Hand searches of references were also conducted to identify other relevant studies.

Data were extracted from the included publications according to the following descriptive categories found in Table 2. These included the location of the intervention; the healthcare professional or other involved in the yarn (for example, if it involved an Aboriginal and/or Torres Strait Islander healthcare professional or non-Indigenous person), which population group was targeted (i.e. health condition and other demographics); how yarning had been used in the health care system (for example, how it was applied—individual or group, face to face or via another medium); and the outcomes reported and the tools used to measure the outcomes (for example qualitative interviews or monitoring of health parameters). Two authors were responsible for the data extraction and analysis. Data were initially extracted by AB and validated and supplemented by RM.

**Table 2:** Data extracted from included publications

Author and year	Location	Overall study objective	Who performed the yarning and training received	Involvement of Community in development of intervention	How the yarning intervention was conducted	Target audience of the yarning intervention	Main topic of focus/health condition addressed.	Method of programme evaluation	Programme outcomes	Other Comments
Begley et al. [12]	Brisbane South Division of General Practice, Queensland	To provide information on health to local Aboriginal community	General practitioners (non-Indigenous) in the area that had expressed interest (*N*=8). Cultural awareness and communication training were provided, and GPs were supplied an Indigenous health resource manual	Inala elders, were involved in selecting topics of interest and reviewing GP training resources	GPs delivered group education sessions in a community setting in their lunch breaks. Yarning was used to improve access to quality health information	Local Aboriginal community members including elders, young women’s groups and parent groups.	General topic areas: common cold, immunisation, women’s health, chronic disease management, and child health.	Self-Report A qualitative and quantitative evaluation is conducted after each topic cycle	Knowledge (100% of participants reported “they learnt something”). Satisfaction and understanding (85% reporting ease of understanding based on yarning format). GP’s reported improved understanding of Indigenous community, communication, holistic health and importance of family.	This intervention was reported as an ongoing initiative and improved knowledge and programme satisfaction
Fletcher et al. [13]	Victorian Aboriginal Community Controlled Health Organisation. (VACCHO)	To develop an inclusive policy around smoking habits for workers in the VACCHO.	The Project Officer who conducted yarns was an Aboriginal staff member of VACCHO. Details re-training were not reported	Aboriginal staff were involved in this participatory action research at both the development and implementation stages.	The intervention was conducted in phases. These included drop-in sessions for all VACCO workers, informal yarns in corridors and meeting places where smoking was common, and yarning sessions with managers after policy development.	All VACCHO staff members were involved in order to start conversations about smoking and produce a smoking policy for VACCHO	Smoking	Personal views around smoking habits and smoking consequence were qualitatively gathered during yarning sessions. A participatory action research framework was used to develop policy.	This programme resulted in policy development that banned smoking within all VACCHO buildings and vehicles, and within 3 m of air vents or within 3 m of all entrances and exits of the buildings. Many staff also reported wanting to give up smoking and support was imbedded into policy.	The intervention described resulted in a new policy, rather than having a focus on individual outcomes. Indirect impact on health however was reported.
Dimer et al. [14]	Metropolitan Aboriginal Medical Service (AMS) in Western Australia	To improve cardiovascular health	Staff at the AMS provided the intervention (not stated if Indigenous) Details of training were not reported	Focus groups with Aboriginal health professionals and community members were conducted prior to programme implementation to ensure it would meet community needs and expectations.	Yarning was used to deliver education about cardiovascular disease including diet, exercise, medications, risk factors. This was provided alongside an exercise-based intervention. The clinic was run each Thursday from 9am-1pm, and participants could come at any time within this timeframe with a flexible approach to attendance rather than an appointment-based system.	Aboriginal people were referred by a medical practitioner or self-referred based on high cardiovascular risk. 64% of participants were female.	Cardiovascular health	Mixed methods were employed to evaluate the outcomes of the programme. These included interviews, questionnaires and yarning sessions as well as objective assessment of cardiovascular risk factors. Changes in risk factors were evaluated pre- and post-programme using paired *t* tests. *P* < 0.05 was accepted for statistical significance	Twenty-eight participants who attended at least 8 weeks of sessions achieved a significant decrease in BMI, waist girth, blood pressure, and an increase in 6-min walking distance. Qualitative consultation revealed strong support for the programme.	The flexibility of the intervention offered was perceived as more culturally appropriate. Participation increased during the study period. The yarning outcomes of improvements in physiological health parameters cannot be separated from the exercise effect.
Crouch [15]	The Loddon Mallee rural region of Victoria	To develop and test a community-led resource to support and empower parents to improve health behaviours	The interviewer was a female full-time Malle District Aboriginal Service (MDAS) clinician of Anglo-Saxon heritage Details of training were not reported.	Permission was sought by local elders to conduct the participatory action research study.	21 Aboriginal individuals, families, Elders, professionals and various community members were invited to share their experiences of positive parenting, childhood memories and what children can teach carers. Yarning was used to create an antenatal yarning resource that was written from the perspective of a baby in the womb. This resource was then used with small groups to see how they responded to the tool.	Community members (men, woman and Elders) were involved in the first yarning stages to create the resource. The tool being developed was targeted at Pregnant Aboriginal women.	Antenatal health	Qualitative yarning interviews to create a resource using a participatory action research framework.	The outcomes of the research were the development of a resource for pregnant women to improve their health and the health of their unborn children.	The intervention described resulted in a new resource for pregnant females and their families, rather than have a focus on individual’s outcomes. Indirect impact on health was reported.
Campbell et al. [16]	Nine remote Cape York communities, Queensland	To evaluate the implementation of the Baby One Program (BOP), an Australian family-centred programme for improving child health.	Indigenous health workers from the Apunipma Aboriginal community-controlled health organisation. Details of training were not reported	Not stated	BOP includes 15 visits from health-workers throughout pregnancy and up until the time the child is 2 years and 10months. 7 Baby baskets are delivered with contents of the baskets containing resources for mother and baby. The health worker also has a yarn with the family at each visit with a range of health promotion topics to cover. A programme that started at confirmation of pregnancy and lasted until the baby was 2 years, yarning was used as an information delivery tool	Pregnant women from the time they know they’re pregnant until the baby is 2 years and 10 months	Child health and development	Qualitative evaluation through yarning with staff and families in the community.	The programme was perceived to be useful and necessary by both health workers and family members. Yarning was seen to be beneficial in exchanging information; Information was easier for the mothers to understand, the programme promoted good health through behaviours such as quitting smoking and reducing consumption of alcohol. Health workers reported a reduced risk of families engaging with the Department of Child Safety because of the support provided by the BOP	This study focuses mainly on the implementation of the service rather than health outcomes for children. Improved knowledge was perceived.

### Data analysis and quality appraisal

The data extracted from each of the studies under the framework were then descriptively analysed using an inductive approach to explore which if any participant outcomes were improved and if these improvements were believed to be a direct result of the yarning intervention. No other themes were explored. Both AB and RM analysed each of the publications separately and came to the same conclusions with respect to the outcomes of yarning after discussion. Comments about the outcomes were tabulated in the “Other Comments” section of Table 2.

The quality of included publications was assessed by utilising the appropriate Joanna Briggs Institute (JBI) checklist [17]. These included the checklist for quasi-experimental studies, and the checklist for qualitative research where appropriate. JBI was chosen, as it contains tools for various types of research studies. The authors assigned a quality score of one for every met criterion of the appropriate checklist applied (Appendix 2). This meant that a maximum score of nine was possible. A descriptor of a poor quality was applied to any publication that received a score of 4 or lower, moderate was the descriptor used for publication scoring between 5 and 7 and good was the applied descriptor to those scoring 8 and above. Regardless of quality ranking, no publications were excluded based on their quality assessment.

## Results

**Fig. 1:**
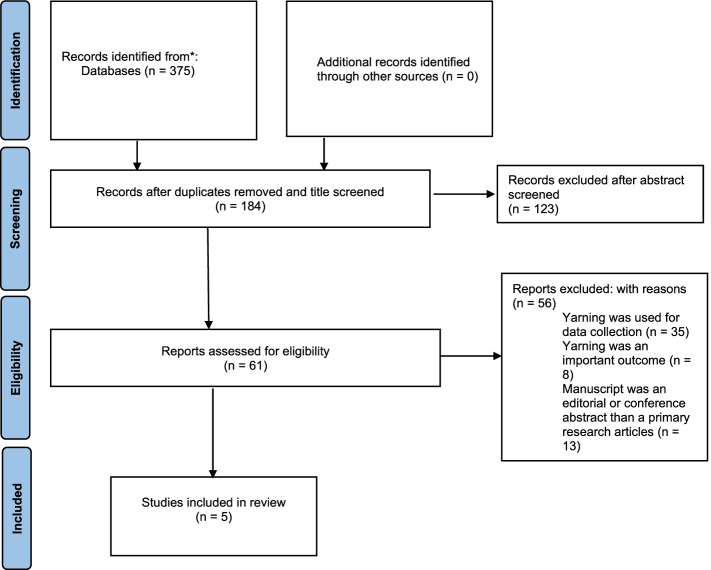
PRISMA flow diagram of included studies

In total, there were 375 papers identified via the database search. After duplicate removal and title screening, 184 abstracts were screened for inclusion. During the abstract review phase, publications were removed where it was apparent that the manuscript was not a primary research article, or yarning was not used as a health intervention. The remaining 61 publications underwent a full-text review where a further 56 publications were removed and no additional publications identified. The majority of those removed at this stage were excluded as the yarning was primarily a data collection tool only and not used as a healthcare intervention (*n*=35). Other reasons for exclusion included publications where yarning was reported as an intervention but was not used as the intervention in the study (*n*=8) or the manuscript was an editorial or conference abstract rather than a primary research article (*n*=13) (Fig. 1). In total, five publications were included in the extraction phase [12–16]. Table 2 provides a description of each individual study including the overall study objective, who conducted the yarning process and the outcome of each study.

The five yarning studies were published between 2005 and 2018. There was a range of health topics that were the focus of the yarning interventions with two out of the five studies focusing on maternal and child health [15, 16]. One focused on a range of health topics [12], one focused on smoking [13] and one on cardiovascular health [14]. In two of the studies, the people providing the yarning intervention were Aboriginal [13, 16]. In two studies, the people conducting the yarning were non-Indigenous [12, 15], and in one study, the nationality of the staff at the Aboriginal Medical Service (AMS) that were involved in the yarning intervention was not stated [14]. The involvement of Aboriginal community in the development of the interventions or the research study was apparent in four out of the five studies [12–15].

Two of the studies were mainly focused on the creation of a resource [15] or policy [13] document and used a participatory action research approach to create these resources that would eventuate as health interventions. Both studies used yarning to gather stories and experiences of participants to empower participants to improve health behaviours by applying a trauma informed lens, such as the damage a mother may cause her unborn child through unhealthy behaviours such as smoking [15]. A powerful quote from the Fletcher et al. study page 95 “we need to talk about why we are doing this; smoking is killing our mob, and this is part of trying to change that” highlights how the participation in creating these resources was also allowing participants to reflect on their own health behaviours, therefore possibly having indirect impact of health outcomes of participants [13]. One study focused on implementation evaluation rather that direct health outcomes [16]. Only two studies therefore aimed to have direct impact on health outcomes [12, 14]. However, the study by Begley et al. only measured self-reported process outcomes such as knowledge and perceptions of the programme rather than actual health outcomes [12]. The study by Dimer et al. was the only study to evaluate physiological outcomes as a result of the intervention [14]. This pre-post evaluation of cardiovascular risk showed significant changes in participants’ health parameters, although the effect of the yarning without the other intervention of exercise cannot be determined [14].

Quality of the included publications was ascertained using the appropriate JBI checklists. The Qualitative research JBI checklist was applied to 4 studies [12, 13, 15, 16] and the Quasi-experimental JBI experimental checklist applied to the Dimer et al. study [14]. The study quality varied from poor to moderate (Appendix 2); however, based on the death of literature, no studies were removed based on quality assessment.

## Discussion

This review focused on the use of yarning as a health intervention. Only five papers out of the 375 found during the initial search had attempted to use clinical yarning as an intervention, and even of those included, only one reported on physiological patient results. The study quality also varied from poor to moderate based on the JBI quality appraisal. Studies showed that yarning was used in a variety of settings and modes which included individual one-on-one yarning or group yarning. It was also used as part of a multimodal intervention, or to create policy or healthcare resources, or as a tool to improve overall public health knowledge. Due to the large variation in studies and the way yarning was used as an intervention and the lack of patient-specific outcomes reported, it is difficult to make any overall conclusions on the impact yarning has on health outcomes.

All included studies in this review had been published within the last 15 years. This may be because focus on Aboriginal and/or Torres Strait Islander health may have gained greater momentum in more recent times. The health disparities between Aboriginal and/or Torres Strait Islander peoples and non-Indigenous Australians was described in the title of a news article from The Age as a “disgrace” [18] around the time of the first publication included in this review [12]. Today, whilst the health statistics for Aboriginal and/or Torres Strait Islander peoples have improved somewhat [2], Australia still has a very long way to go to improving Aboriginal and/or Torres Strait Islander health and healthcare.

Looking at literature from outside Australia, conducted using Indigenous communication strategies in Native American cultures that bare similarities to “yarning” [7, 8], researchers have concluded that there does seem to be a correlation between an Indigenous focus in communication and some improvement of health outcomes [7, 8]. However, it should be noted that due to these being separate cultures, we cannot draw firm conclusions that the same results would occur in Australia hence more research, using a variety of methods in Australia may need to occur.

As culturally appropriate healthcare has been reported to be necessary [19], it may be more appropriate for Aboriginal and/or Torres Strait Islander health professionals to be the healthcare providers for Aboriginal and/or Torres Strait Islander patients. However, the number of Aboriginal and/or Torres Strait Islander health workers are scarce [20], which means that all health professionals may need to be trained to provide culturally appropriate healthcare to this population. In this review, two studies involved non-Indigenous health professionals as the people conducting the yarning [12, 15]. For example, in the study by Begley, local general practitioners were trained to provide clinical yarning on a range of topics [12]. Other studies in the review however did not describe the training provided to the clinical yarners.

The “Yarn with me” resource explores the framework of the clinical yarn and the three fundamental areas that form its framework [5]. It should be noted however that none of the studies in this review referred to this framework nor was it identified or described in any published manuscript. It is not fully clear how the yarning was provided within these studies. In fact, some studies were conducted prior to the release of this framework [12–14]. This framework [5] however may be a useful guide for future clinicians and researchers and may assist in health practitioner training.

Including Aboriginal and/or Torres Strait Islander health in curricula are now mandated accreditation requirements in medical, pharmacy, and nursing schools in Australia [21, 22]. Universities also have graduate attributes that articulate the importance of Aboriginal and Torres Strait Islander cultural awareness and safety and have also recognised that all academic staff should gain knowledge and awareness to assist graduates to obtain these attributes [22]. Recent initiatives have been undertaken to empower health students to open their eyes to the importance of Aboriginal culture and health issues [23, 24]; however, there is still a long way to go to ensure health graduates are ready to provide specialised and tailored services to Aboriginal and/or Torres Strait Islander consumers, such as yarning. A systematic review by Ewen et al. in 2012 identified two studies that had evaluated medical students’ skills in providing culturally appropriate care, and similar to this review, they were unable to conclude that Indigenous health curricula is having any impact on Indigenous health care outcomes [25]. What can be concluded however is that more research and education in this space is required. It should also be noted that whilst learning about and participating in clinical yarning may be part of the journey to becoming a culturally competent practitioner, it is not the full picture. Other aspects to cultural competence include being able to provide a safe space where Aboriginal and/or Torres Strait Islander peoples feel comfortable seeking advice, being able to recognise one’s own personal biases and being able to overcome them and having a background knowledge of the history that Aboriginal and/or Torres Strait Islander peoples went through in the country now called Australia that led to certain outcomes today.

The studies included in this review were not established to be of high quality after the application of the appropriate JBI checklist. No conclusive data were gathered to prove the effectiveness of clinical yarning, and none employed high-quality design to assess this outcome [26]. In fact, the studies by Fletcher et al. [13] and Crouch et al. [15] were borderline in whether clinical yarning was in fact used as an intervention and received a moderate score in their quality appraisal. As stated however, though yarning was used primarily as a method to create a resource or policy, these studies in fact resulted in behaviour changes for some involved in these studies. For these reasons, in comparison to the other excluded studies these two studies which involved yarning for a “clinical” purpose were included in this review.

Only one study reported on actual physiological outcomes [14] while others reported on other process evaluation outcomes only [12, 13, 15, 16]. The study by Dimer et al. [14] used both yarning and exercise as the intervention to improve cardiovascular risk factors, and because of the multimodal intervention and the lack of a control group, it is difficult to make a determination if the yarning added to improvements in patient outcomes. In saying that, it may not be culturally appropriate and hence ethical to undertake more rigorous clinical trials in this area to prove that yarning indeed has impact on health outcomes. Further, future studies may also look to gather patient perspectives of clinical yarning interventions in a more qualitative manner to draw inferences of benefit. Despite the lack of findings of clinical yarning’s impact, it is apparent that none of the publications stated that clinical yarning would be a detriment to healthcare and health communication. Hence, it does appear that it is a well-received way to provide health information to Aboriginal and/or Torres Strait Islander patients. Some papers cite the use of clinical and research yarning is a good method to break down barriers and walls of communication that would generally be present when using standard methods of communication [5, 27]. Due to centuries of the First Nations People being treated in the traditional western paternalistic style of health care, implementation of this more culturally appropriate style of care could be seen as an olive branch in trying to address the issues that have for been affecting these communities for years. Future studies of the benefits of using a variety of techniques could occur simultaneously as this becomes more widespread in practice and policy.

From the included studies therefore, inference can be made that if yarning was widely used as a healthcare intervention in this population group, health outcomes may be improved. In fact, the study by Dimer et al. noted that over the duration of the Cardiac Rehabilitation Service, patient attendance rates increased [14]. This is important because it points to the hypothesis that if culturally competent healthcare delivery is implemented, it may be possible to facilitate greater interest in one’s healthcare and encourage Aboriginal and/or Torres Strait Islander peoples to actively take part in their healthcare.

The main strengths of this review include the wide array of databases that were used to undertake the search and that the lead author was able to review the studies with an Aboriginal lens, as he is a proud Wiradjuri man. Further, the validation of the database yields and data extraction from included publications was provided by a second author. The review however is not without limitations. As the reviewers restricted the search strategy to “yarning” only, articles may have been missed describing communications with Aboriginal and/or Torres Strait Islander peoples that chose not to use this terminology. Further, by only including publications published as primary research articles, some articles may have missed other reported outcomes of clinical yarning that could be found in the grey literature such as conference abstracts or unpublished research reports.

## Conclusion

Whilst clinical yarning may be an appropriate way to provide care to Aboriginal and/or Torres Strait Islander peoples, more research is needed in this area due to the scarcity of research. This review showed a range of ways that clinical yarning had been utilised as a healthcare intervention but did not allow for any clear conclusions to be made regarding its impact on health outcomes.

## Data Availability

Full texts of included publications are publicly available.

## Appendix 1

Table 3

**Table 3:** Database Yield with the single word “yarning” search strategy

Database	Extracted papers
Scopus	116
Embase	87
CINAHL	49
MEDLINE	61
IPA	0
APAIS-Health	47
ATSI-Health	15

## Appendix 2

### Quality appraisal rankings

Table 4

**Table 4:** JBI qualitative research checklist

	Is there congruity between the stated philosophical perspective and the research methodology?	Is there congruity between the research methodology and the research question or objectives?	Is there congruity between the research methodology and the methods used to collect data?	Is there congruity between the research methodology and the representation and analysis of data?	Is there congruity between the research methodology and the interpretation of results?	Is there a statement locating the researcher culturally or theoretically?	Is the influence of the researcher on the research, and vice- versa, addressed?	Is the research ethical according to current criteria or, for recent studies, and is there evidence of ethical approval by an appropriate body?	Do the conclusions drawn in the research report flow from the analysis, or interpretation, of the data?	Author quality rank
Begley et al.	U	Y	Y	U	Y	U	N	U	Y	Poor
Fletcher et al.	Y	Y	Y	U	Y	Y	Y	U	Y	Moderate
Crouch	U	Y	Y	Y	Y	Y	N	N	Y	Moderate
Campbell	U	Y	Y	Y	Y	N	N	Y	Y	Moderate

Table 5

**Table 5:** JBI quasi-experimental JBI checklist

	Is it clear in the study what is the cause’ and what is the “effect” (i.e., there is no confusion about which variable comes first)?	Were the participants included in any comparisons similar?	Were the participants included in any comparisons receiving similar treatment/care, other than the exposure or intervention of interest?	Was there a control group?	Were there multiple measurements of the outcome both pre- and post-intervention/exposure?	Was follow up complete and if not, were differences between groups in terms of their follow up adequately described and analysed?	Were the outcomes of participants included in any comparisons measured in the same way?	Were outcomes measured in a reliable way?	Was appropriate statistical analysis used?	Author quality rank
Dimer et al.	Y	Y	Y	N	N	Y	Y	Y	Y	Moderate

JBI checklist legend (*Y* yes, *N* no, *U* unsure, *NA* not applicable)
